# Natural Biomolecule Ovomucin–Chitosan Oligosaccharide Self-Assembly Nanogel for Lutein Application Enhancement: Characterization, Environmental Stability and Bioavailability

**DOI:** 10.3390/jfb15040111

**Published:** 2024-04-21

**Authors:** Qi Xu, Haoye Teng, Xuanchen Li, Zhenqing Zhang, Yumeng Han, Haixin Sun

**Affiliations:** 1 College of Life Science, Qingdao University, Qingdao 266000, China; hy12122021@163.com; 2 Institute of Advanced Cross-Field Science, Qingdao University, Qingdao 266000, China; lxc1559360735@163.com (X.L.); zhangzhenqing0330@163.com (Z.Z.); hanym0105@163.com (Y.H.)

**Keywords:** nanogel, lutein, stability, bioavailability, oral delivery

## Abstract

As an essential nutrient, lutein (LUT) has the ability to aid in the prevention of eye diseases, cardiovascular diseases, and cancer. However, the application of LUT is largely restricted by its poor solubility and susceptibility to oxidative degradation. Thus, in this study, LUT-loaded nanogel (OVM-COS-LUT) was prepared by a self-assembly of ovomucin (OVM) and chitosan oligosaccharide (COS) to enhance the effective protection and bioavailability of LUT. The nanogel had excellent dispersion (PDI = 0.25) and an 89.96% LUT encapsulation rate. XRD crystal structure analysis confirmed that the encapsulated LUT maintained an amorphous morphology. In addition, the nanogel showed satisfactory stability with pH levels ranging from 2 to 9 and high ionic strengths (>100 mM). Even under long-term storage, the nanogel maintained an optimistic stabilization and protection capacity; its effective retention rates could reach 96.54%. In vitro, digestion simulation showed that the bioaccessibility and sustained release of OVM-COS-LUT nanogel was superior to that of free LUT. The nanogel provided significant antioxidant activity, and no significant harmful effects were detected in cytotoxicity analyses at higher concentrations. In summary, OVM-COS-LUT can be utilized as a potential safe oral and functional carrier for encapsulating LUT.

## 1. Introduction

Carotenoids are one of the essential nutrients for physiological health [1]. As a member of the carotenoid family, LUT has shown benefits in preventing diseases and easing symptoms, such as reducing the risk of cataracts or age-related macular degeneration [2,3], cancer prevention [4], and immunomodulation [5].

However, natural carotenoids are found only in plants or microorganisms. For humans, uptake through daily ingestion rather than synthesis is the only way to obtain LUT [6]. Although foods like corn are an abundant source of LUT, its poor water solubility limits its bioavailability via oral administration [7]. In addition, its unique chemical structure makes LUT sensitive to light, heat, and oxygen. Therefore, the oral application of LUT in medicine and nutritional supplements faces severe challenges [8].

One of the current strategies to solve this problem is to encapsulate bioactive substances into nano-delivery systems [9]. There is great potential to utilize nanomaterials’ large surface area-to-volume ratio to improve bioavailability, controlled release, and sensory improvement of active ingredients [10]. There are two main methods for generating nanoparticle systems; one is a “top-down” approach where larger-sized materials are dispersed and milled [11]. This method is prone to raw material waste and requires higher energy support. The other is the “bottom-up” approach, where small molecules are chemically synthesized or self-assembled into nanostructures [11]. Among them, self-assembly from natural biomolecules, especially proteins or polysaccharides, possesses advantages for the design or manufacture of nanocarriers [12]. Those nanocarriers become catabolized and absorbed as nutrients after entering the digestive tract; therefore, they have low immunogenicity and high biocompatibility [13]. In addition, without using other chemical cross-linking agents, natural protein–polysaccharide nano-systems can self-assemble through electrostatic interactions and hydrogen bonding, which is also more eco-friendly and safer. Ma et al. prepared nanoemulsion loaded with β-carotene using soy protein isolate (SPI) and citrus pectin (CP), and the ultrasound-treated group showed excellent storage stability [14]. Li et al. encapsulated lutein using zein-soluble soybean polysaccharide composite nanoparticles, and the bioaccessibility of encapsulated lutein was significantly higher than that of the free group [15].

OVM, an anionic protein isolated from eggs, consists of low-carbohydrate α1- subunits, α2-subunits, and high-carbohydrate β-subunits polymerized by disulfide bonds [16]. OVM has a variety of functional activities, such as antiviral [17], anti-tumor [18], and anti-inflammatory [19] effects. The application of OVM as a novel functional component in nano-delivery systems is yet to be explored. Due to its unique molecular structure, OVM has excellent gelling properties, which makes it a potential material for constructing nanogel [20]. The natural amphiphilicity of OVM also provides sufficient hydrophobic space for the payload of hydrophobic substances. In addition, to enhance the stability of the protein delivery body after encapsulating the cargo, a low molecular weight in COS was utilized to induce the construction further. COS, a cationic polysaccharide derived from chitin/chitosan, has good biocompatibility, water solubility, and adhesion [21], and can be self-assembled by electrostatic force binding to OVM.

Given the factors above, OVM and COS were used to prepare lutein-coated oral-delivery-based nanogel. A structure analysis showed that all the components bonded to each other through self-assembly during the formation of nanogel. The nanogel exhibited a wide pH range, ionic strength, and long-term storage stability. Meanwhile, LUT’s antioxidant ability and bioavailability were raised through binding with nanogel. The biocompatibility of nanogel was also verified by a cytotoxicity assay. In summary, this study provides new insights into the nano-delivery applications of OVM-COS, offering a potential strategy for effectively utilizing hydrophobic LUT.

## 2. Materials and Methods

### 2.1. Materials

Lutein (purity > 90%), chitosan oligosaccharide (molecular weight < 2000), ethyl acetate, 1,1-diphenyl-2-pyridine-hydrazyl radical (DPPH), methanol, and acetonitrile for HPLC were purchased from Macklin Biochemical Co., Ltd. (Shanghai, China). All other chemicals were of analytical grade.

Ovomucin (purity > 90%) was extracted from fresh eggs by a two-step salt ion precipitation method [22]. The extracted ovomucin was ultrasound-treated (Scientz, Ningbo, China), and after centrifugation (15,000 rpm, 4 °C, 30 min), the supernatant was separated to obtain the ovomucin solution.

### 2.2. Preparation of OVM-COS and OVM-COS-LUT Nanogel

Under stirring at 200 rpm, 500 μL of an LUT solution (2 mg/mL, DMSO) was slowly added to a 10 mL OVM solution (1.25 mg/mL), and the mixture was ultrasound-treated (100 W, On/Off 5 s) in an ice bath for 5 min. Then, 500 μL of COS solution (1 mg/mL) was slowly added to the above mixture, and stirring was continued at 200 rpm for 60 min. The solution was slightly centrifuged (5000× *g*, 10 min) to remove insoluble matter and then transferred to a dialysis bag for 12 h to remove DMSO. The prepared nanogel was stored at 4 °C for further research. Meanwhile, OVM-COS nanogel without the addition of LUT was prepared.

### 2.3. Determination of Average Particle Size, Zeta Potential, and PDI

Determination was performed using Zetasizer Nano (Malvern Instruments Corp., Malvern, UK). Before measurement, the nanogel samples were diluted with deionized water to a protein concentration of 0.5 mg/mL and equilibrated at 25 °C for 2 min.

### 2.4. Encapsulation Efficiency (EE) and Loading Capacity (LC) of Lutein

The EE and LC of LUT by OVM-COS-LUT nanogel were determined by ethyl acetate extraction [23]. Utilizing the insolubility of ethyl acetate with the aqueous phase, the free lutein in the aqueous phase nanogel was effectively extracted. Briefly, 1 mL of LUT-loaded nanogel was mixed with 3 mL of ethyl acetate. The mixture was vortexed vigorously for 30 s and centrifuged at 5000 rpm for 5 min. The supernatant phase of the mixture was separated, and the absorbance of free lutein dissolved in ethyl acetate was measured at 448 nm using a UV–visible spectrophotometer (UV-2600, Shimadzu, Kyoto, Japan). A standard curve was established for the standard lutein solution in 0–20 μg/mL (y = 0.065x − 0.0049, R^2^ = 0.9999). The encapsulation efficiency (EE) and loading capacity (LC) of lutein was calculated according to the following equation:

(1) $$\mathrm{EE}\left(\%\right)=\frac{\mathrm{Total}\;\mathrm{lutein}\;\mathrm{content}-\mathrm{Free}\;\mathrm{lutein}}{\mathrm{Total}\;\mathrm{lutein}\;\mathrm{content}}\times 100\%$$

(2) $$\mathrm{LC}\left(\%\right)=\frac{\mathrm{Total}\;\mathrm{lutein}\;\mathrm{content}-\mathrm{Free}\;\mathrm{lutein}}{\mathrm{Total}\;\mathrm{nanogel}\;\mathrm{content}}\times 100\%$$

### 2.5. Morphology Analysis

The morphology of OVM-COS-LUT nanogel was observed using a transmission electron microscope (HT7700, Hitachi Ltd., Tokyo, Japan). A total of 20 μL of the newly prepared sample was dropped on a 200-mesh carbon-coated copper grid. After 5 min, excess samples were removed from the copper grid with filter paper and dried naturally at room temperature. Samples were observed under 80 kV accelerated voltage.

### 2.6. Ultraviolet–Visible (UV–Vis) Spectroscopy

The UV–visible spectra of OVM-COS and OVM-COS-LUT nanogel dispersion were measured using a UV–visible spectrophotometer. Wavelength scans were observed in a range of 300–700 nm.

### 2.7. Fluorescence Spectroscopy

Protein endogenous fluorescence spectra of OVM, OVM-COS, and OVM-COS-LUT were measured using a fluorescence spectrophotometer (Spectrofluorometer FS5, Edinburgh Instruments, Livingston, UK). The sample protein concentration was diluted to 0.5 mg/mL; the excitation wavelength was 274 nm. The wavelength scan range was 280–500 nm; the excitation and emission slit widths were set to 2 nm.

### 2.8. Circular Dichroism (CD) Spectroscopy

Circular dichroism spectra of OVM, OVM-COS, and OVM-COS-LUT were measured using a circular dichroism spectrometer (J-810, JASCO, Tokyo, Japan). Sample protein concentrations were diluted to 0.3 mg/mL, temperature to 25 °C, quartz cell optical path length to 0.1 cm, wavelength scan range to 190–240 nm, speed to 10 nm/s, and resolution to 0.1 nm.

### 2.9. Fourier-Transform Infrared (FTIR) Spectroscopy

The infrared spectra of LUT, COS, OVM, OVM-COS, and OVM-COS-LUT powders were determined using a Fourier-Transform Infrared (FTIR) spectrophotometer (Nicolet iS50, Thermo Fisher Scientific Inc., Madison, WI, USA). The powder samples were pressed into flakes at room temperature. Then, 32 scans were repeated, and spectra were recorded in the range of 4000–500 cm^−1^.

### 2.10. X-ray diffraction (XRD)

X-ray diffraction spectra of LUT, OVM, COS, OVM-COS, and OVM-COS-LUT powders were determined using an X-ray diffractometer (XRD) (Smart Lab 3KW, Rigaku Inc., Tokyo, Japan) with a test voltage of 40 kV, a current of 40 mA, a 2θ range of 10–30°, and a speed of 5°/min.

### 2.11. Stability Analysis 

#### 2.11.1. pH Stability

Freshly prepared OVM-COS-LUT nanogel was mixed with twice the volume of deionized water. The samples were then adjusted to the desired pH (2–9) with 0.1 mol/L NaOH or HCl. After 6 h of standing, the average particle size, PDI, and zeta potential of the OVM-COS-LUT nanogel were measured at different pH environments. Before measurement, the sample was equilibrated at 25 °C for 2 min.

#### 2.11.2. Ionic Strength Stability

The pH environment of the newly prepared OVM-COS-LUT nanogel was adjusted to 6 with 0.1 mol/L HCL and then mixed with equal volumes of different concentrations of NaCl solutions (pH = 6), with the final NaCl concentrations being 0, 50, 100, 200, 300, 400, and 500 mM. After 6 h of standing, the average particle size and PDI of the OVM-COS-LUT nanogel were measured at different ionic strengths. Before measurement, the sample was equilibrated at 25 °C for 2 min.

#### 2.11.3. Storage Stability

Freshly prepared OVM-COS-LUT nanogel was stored at 4 °C for 30 days. Every 5 days, equal amounts of nanogel were aspirated, and the average particle size and PDI of the OVM-COS-LUT nanogel were measured. Before measurement, the sample was equilibrated at 25 °C for 2 min. The UV absorption intensity of the nanogel was measured by a UV–Vis spectrophotometer. Changes in the nanogel were observed by optical photo documentation.

### 2.12. Antioxidant Activity 

The antioxidant capacity of free LUT, OVM-COS, and OVM-COS-LUT nanogel was determined using the DPPH radical scavenging ability [24]. First, 1 mL of different sample concentrations was homogenously mixed with 2.0 mL of DPPH (0.02 mM), and the reaction was carried out at 25 °C for 30 min under light-proof conditions. The absorbance of the samples was measured at 517 nm. Ethanol was used in place of the DPPH solution for blanks, and deionized water was used in place of the sample solution for controls. The DPPH radical scavenging rate was calculated according to the following equation:

(3) $$\mathrm{DPPH}\;\mathrm{radical}\;\mathrm{scavenging}\;\mathrm{capacity}\left(\%\right)=1-\frac{{(A}_{1}-A_{2})}{A_{0}}\times 100\%$$

A_1_ represents the absorbance of the sample group; A_2_ and A_0_ represent the absorbance of the blank and control groups, respectively.

### 2.13. In Vitro Digestion Behavior

#### 2.13.1. Bioaccessibility

An in vitro digestion method suitable for food standardization to evaluate the bioaccessibility of LUT was referenced [25].

Simulated gastric fluid (SGF): 10 mL fresh OVM-COS-LUT nanogel was mixed with 10 mL of simulated gastric fluid (SGF, 1 mg/mL pepsin). The 20 mL mixture was adjusted to pH 2.0 with 0.5 mol/L HCl and incubated in a shaker at 100 rpm at 37 °C for 2 h.

Small intestinal fluid (SIF): The above gastric mock mixture was adjusted to pH 7.0 with 0.5 mol/L NaOH to inactivate the pepsin. Trypsin was added to the 20 mL mixture to simulate the small intestinal fluid (SIF, 2 mg/mL trypsin). The mixture was then incubated in a shaker at 100 rpm at 37 °C for 3 h.

At the end of gastric and intestinal digestion, 1 mL of each reaction mixture was centrifuged at 10,000× *g* for 30 min. After centrifugation, the supernatant was filtered through a 0.45 μm filter to obtain soluble LUT. The filtrate was diluted in ethanol absolute and passed through a 0.22 μm filter membrane, and LUT release was determined by high-performance liquid chromatography (HPLC) [26]. For free LUT, it was treated using the same digestion simulation described above. The in vitro bioaccessibility of LUT was calculated according to the following equation:

(4) $$\mathrm{Bioaccessibility}\left(\%\right)=\frac{A_{0}}{A_{1}}\times 100\%$$

where A_0_ represents the weight of LUT in the supernatant after simulated gastric or intestinal digestion, and A_1_ represents the initial weight of LUT before simulated digestion.

HPLC analysis was performed on an Agilent 1260 liquid chromatography system (1260 Infinity II, Agilent Technologies Inc., Santa Clara, CA, USA) equipped with an SB-C18 HPLC column (4.6 mm, 250 mm, 5 μm). The mobile phase was 90% acetonitrile + 10% methanol at a flow rate of 1 mL/min, with an injection volume of 20 μL, detection at 470 nm, and a column temperature of 25 °C. The standard curve was made with 2–10 μg/mL of ethanolic solution of LUT (y = 62.391x + 7.1939, R^2^ = 0.9973).

The average particle size of the nanogel was determined by Zetasizer Nano during the simulation of gastrointestinal digestion.

#### 2.13.2. In Vitro Release

To create conditions for hydrophobic LUT release, the release medium consisted of a 50% gastrointestinal simulating solution mixed with 50% anhydrous ethanol [27]. A total of 5 mL of OVM-COS-LUT nanogel or LUT was placed into a dialysis bag, after which the airtight dialysis bag was placed into a release medium containing 100 mL of SGF (pH = 2, 0.5 mg/mL pepsin) and then incubated for 2 h in a shaker at 100 rpm, 37 °C. The dialysis bag was then transferred to another one containing 100 mL of an SIF (pH = 7, 1 mg/mL trypsin) release medium, and the incubation was continued for 3 h. The amount of lutein released was determined using a UV–visible spectrophotometer by aspirating 5 mL of the release medium every 30 min, and an equal volume of the release medium was replenished. The release rate of lutein was calculated according to the following equation:

(5) $$\mathrm{Release}\;\mathrm{rate}\left(\%\right)=\frac{C_{0}}{C_{1}}\times 100\%$$

C_0_ is the concentration of LUT in the release medium after a certain time interval, and C_1_ is the initial concentration of LUT in the dialysis bag.

### 2.14. Cytotoxicity Analysis

The effect of OVM-COS-LUT nanogel on the activity of L929 cells (Shanghai Jining Industrial Co., Ltd., Shanghai, China) was determined using the MTT (3-(4,5-dimethylthiazol-2)-2,5-diphenyltetrazolium bromide salt) method [28]. Cells were cultured in DMEM containing 10% fetal bovine serum, 100 μg/mL penicillin, and 100 μg/mL streptomycin at 37 °C in a 5% CO_2_ humidified environment. L929 cells were inoculated in 96-well plates at 8 × 10^3^ cells/well cell density. The cell suspension was 80 μL/well and incubated at 37 °C for 24 h. Then, 20 μL of OVM-COS-LUT nanogel at different concentrations was added to the 96-well plates. The final concentration of nanogel in the cell culture mixture was 0, 25, 50, 75, 100, 150, and 200 μg/mL, and the final volume of cell suspension was 100 μL/well. After 24 h of incubation, 40 μL of an MTT solution was added to each well, and incubation continued for 4 h. After incubation, the medium was removed, 150 μL of dimethyl sulfoxide (DMSO) was added to each well, and the absorbance was measured at 492 nm after incubation for 30 min in the dark.

### 2.15. Data Analysis

Analysis of variance (ANOVA) was performed using IBM SPSS Statistical version 26.0 software. Data were processed with Origin 2018 software. Results were expressed as mean ± SD value of three replicates.

## 3. Results and Discussion

### 3.1. Characterization of Nanogel

Table 1 shows the characteristics of OVM, COS, OVM-COS, and OVM-COS-LUT, including size, PDI, zeta potential, EE, and LC. OVM carried a plentiful negative charge due to the presence of groups such as sialic acid or glycan sulfate. As a cationic polysaccharide, COS had the property of carrying positively charged groups. Compared with OVM, the ζ-potential of OVM-COS was altered, demonstrating the existence of electrostatic interactions between COS and OVM. In addition, COS did not have the characteristics of a colloidal particle with a PDI of 1. However, COS could act as a small molecular weight inducer, which induced a more homogeneous system (PDI 0.44–0.29) of OVM-COS with only a slight increase in average particle size. Generally, it can be considered a homogeneous system when PDI is below 0.3 [29].

The electrostatic repulsion force was an important element in the stability of nano-systems. The surface charge of colloidal particles affects the strength of the intermolecular electrostatic repulsion, and too low an electrostatic repulsion can cause aggregation between colloidal particles [30]. The interaction of COS with OVM reduced the surface charge of the nanogel, but it still exhibited the highly negative charge characteristic of OVM, indicating that low molecular weight COS may have been interspersed between OVM during the nanogel formation, thus facilitating the cross-linking between the components. The large charges gathered by OVM on the nanogel surface could still stabilize the nanogel through the formation of electrostatic repulsion between the particles. In addition, as a similar phenomenon described in a previous study [15], the particle size increased significantly (127.33 ± 2.01 to 209.16 ± 1.71 nm) after LUT loading. The interaction of LUT with OVM-COS had no influence on the homogeneity of OVM-COS-LUT (PDI = 0.25). Moreover, the high encapsulation efficiency (89.96 ± 0.32%) proved that the nanogel provided a strong loading capacity for LUT.

**Figure 1:**
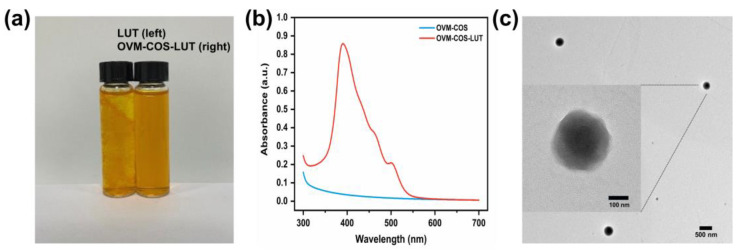
Optical pictures of LUT and OVM-COS-LUT (**a**). UV–Vis spectrum of OVM-COS and OVM-COS-LUT, ranging 300–700 nm (**b**). TEM image (scale bar = 100/500 nm) of OVM-COS-LUT (**c**).

As shown in Figure 1, The macro and micro forms of OVM-COS-LUT were investigated. OVM-COS-LUT presented itself as a brownish-yellow solution with low scattering (Figure 1a). Due to the low water solubility of LUT, the LUT solution showed aggregation. The result showed that LUT was stably dispersed in the solution by nanogel encapsulation. Compared to OVM-COS, OVM-COS-LUT showed a strong absorption peak in UV–Vis spectra around 390 nm (Figure 1b). The TEM morphology of the OVM-COS-LUT nanogel is shown in Figure 1c. Colloidal particles in the image were approximately spherical with a size of about 200 nm, consistent with DLS measurement. Furthermore, the shadow in the center and the surrounding light halo indicated that the colloidal particles had a characteristic core–shell structure.

### 3.2. Structure Analysis

**Figure 2:**
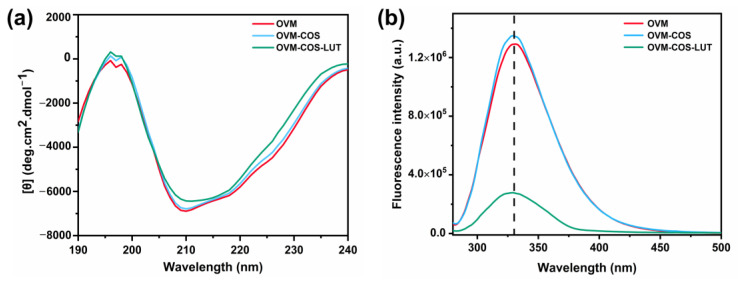
Circular dichroism (CD) spectra (**a**) and fluorescence spectra (**b**) of OVM, OVM-COS, and OVM-COS-LUT.

Circular dichroism is a rapid and accurate method to determine the changes in secondary structure [31]. The CD spectra of OVM, OVM-COS, and OVM-COS-LUT nanogel are shown in Figure 2a, and the percentage of secondary structure is listed in Table 2. For each sample, the negative bands near 210 nm and 218 nm were ascribed to the α-helix, and the positive bands at 196 nm and 198 nm were ascribed to the β-sheet [31]. As shown in Table 2, COS hardly changed the α-helix, but the content of the β-sheet increased. The main reason causing this phenomenon was attributed to the electrostatic attraction between OVM and COS [32,33]. An increase in the β-sheet usually appeared with protein aggregation [34]. Those results corresponded with the scene in which COS gathered OVM molecules through electrostatic interaction. For LUT-loaded nanogel, the upward shift in spectrum near 210 nm signified a decrease in the α-helix, amounting to 4.56%. Nevertheless, the content of the β-sheet was further expanded to 43.53%, induced by LUT. The transformation could be attributed to the changes in hydrogen bonds and the hydrophobic interaction of OVM, eventually leading to recombination in the secondary structure.

The formation of nanogel is also coupled with changes in tertiary structure. Figure 2b shows the fluorescence tendency of OVM, OVM-COS, and OVM-COS-LUT using tyrosine as a probe. An increase or decline in fluorescence generally accompanies structure transformation in protein. The conformation and folding state of OVM were changed in OVM-COS formation, resulting in changes in intrinsic fluorescence intensity. The fluorescence peak shift was considered the signal representing microenvironment changes to the amino acid. As shown in Figure 2b, no band shift was exhibited in the spectrum before LUT addition, but a blue shift was exhibited in OVM-COS-LUT (329 to 323 nm). Some regions within OVM, especially hydrophobic ones, could curl inward in LUT binding. Hence, the tyrosine residues entered a hydrophobic environment, leading to a peak shift in nanogel [35]. In addition, with the addition of LUT, the fluorescence intensity of OVM decreased sharply. The quenching effect further indicated that the conformation of OVM was altered by the interaction between components.

### 3.3. FT-IR and XRD Analysis

**Figure 3:**
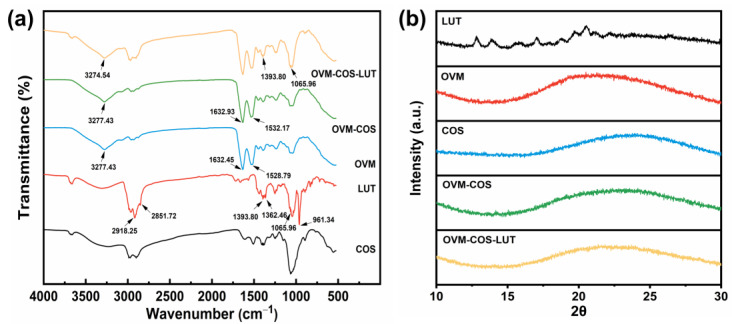
FT-IR spectra (**a**) and XRD spectra (**b**) of COS, LUT, OVM, OVM-COS, and OVM-COS-LUT.

FT-IR spectroscopy is a classical method in interaction studies. The FT-IR bands of all samples are shown in Figure 3a. Free LUT showed several characteristic peaks in a wide range, including the stretch vibration peak of -C-H (2918.25 cm^−1^ and 2851.72 cm^−1^), the peak of the dimethyl splitting group (1362.46 cm^−1^) or trans-conjugated alkene -CH=CH- (961.34 cm^−1^), and some others [36,37]. Remarkably, compared with OVM-COS, the presence of LUT characteristic peaks in LUT-OVM-COS indicated successful LUT encapsulation. The wide band between 3600 and 3200 cm^−1^ could be assigned to the stretch vibration peak of -OH [38]. The peak shifted from 3277.43 cm^−1^ to 3274.54 cm^−1^ after OVM-COS-LUT formation, indicating a hydrogen bond linking LUT to OVM. Indeed, the amide bands shift was evidence of the electrostatic interaction between protein and polysaccharide [39]. After the addition of COS, the amide I and amide II bands in OVM were shifted from 1632.45 to 1632.93 cm^−1^ and 1528.79 to 1532.17 cm^−1^. Therefore, electrostatic interaction was likely the main driving force for the colloidal particle formation between OVM and COS. All these results supported these inferences; nanogel was formed by electrostatic force, and LUT was anchored in the system by hydrogen bonding.

The form of function molecules determines their biological activity to some extent. The crystal structure is an essential factor in this aspect. As shown in Figure 3b, the crystal form of all samples was measured by XRD. For the band of LUT, several strong diffraction peaks were found in the range over 10–25°, indicating a high degree of crystallinity. On the contrary, COS and OVM only exhibited an amorphous broadband. Meanwhile, the effect of COS on OVM was just peak shifting. Those phenomena indicated that polysaccharide derivatives, proteins, or their complexes were amorphous structures. However, no crystal diffraction peaks of LUTs were found in OVM-COS-LUT. The interaction between LUT and the nanocarrier might be the primary cause of crystalline state transformation [40]. In addition, amorphous nutrients were considered as holding better bioavailability [41]. Therefore, lutein-loaded nanogel may lead to better absorption or acceptability in oral administration.

### 3.4. pH Stability Analysis

**Figure 4:**
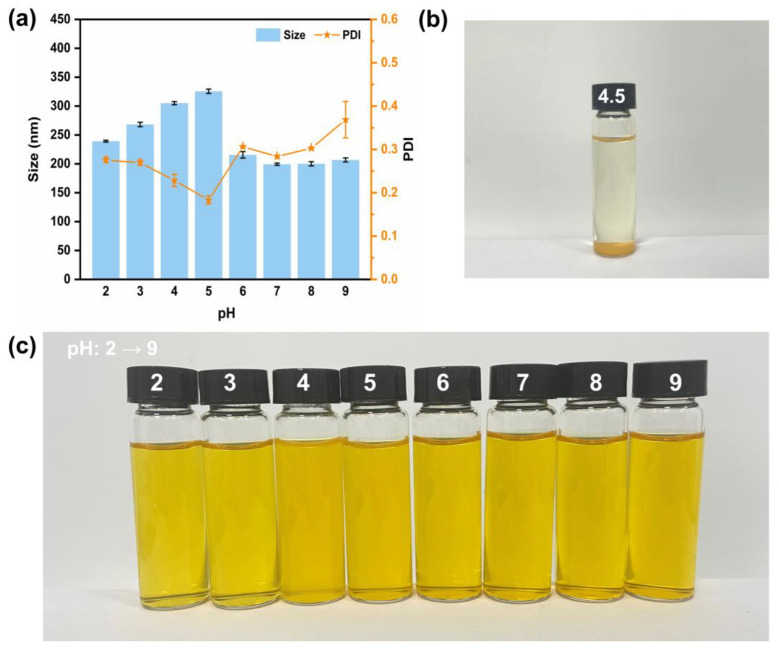
Particle size, PDI of OVM-COS-LUT at pH 2–9 (**a**). Optical image of OVM-COS-LUT at pH = 4.5 (**b**). Optical images of OVM-COS-LUT at different pH 2–9 (**c**).

When ingested by the body, nanogel faces a complex digestive environment, especially floating pH. Thus, investigating the stability of nanogel at different pH levels is essential. Figure 4a shows the particle size and PDI of nanogel at pH 2–9. In the beginning, the size of the nanogel increased with pH 2 to 5. When the pH was 6–9, the particle size was maintained at around 200 nm. Under acidic conditions (pH ≤ 3), OVM and COS maintain the same surface charge, which may cause repulsion between the two components, manifested as a slight swelling of the colloidal particles. Furthermore, particle aggregation at pH = 4 or 5 (near the OVM isoelectric point) resulted in a larger average size. However, this aggregation might result in a decrease in smaller particles, exhibiting lower PDI.

As analyzed by the nanogel construction, the external characteristics of OVM-COS-LUT with a core–shell structure was closer to the OVM shell. Thus, the nanogel was altered to sediment only when the pH was the isoelectric point of OVM (about 4.5, Figure 4b). The strength of the surface charge of the particles can reflect the magnitude of the inter-particle electrostatic repulsion, and a smaller electrostatic repulsion may not be able to maintain the inter-particle stability. As shown in Table 3, at pH 4.5, the electrostatic charge of the particles only wrapped −3.1 mv, resulting in a decline of electrostatic repulsion, which made it hard to prevent the particles from approaching each other. Finally, the particles gradually aggregated to form precipitation. In an environment that gradually deviates from the OVM isoelectric point, the surface charge of the particles gradually increases, maintaining the stability of the system. As humans’ dominant digestion and absorption area, the small intestine was responsible for most LUT absorption. This specific scenario should be considered here. Although the size fluctuated, the nanogel maintained its stability under acidic pH. In addition, a related study has confirmed that OVM can resist protease to a certain extent [16]. It is reasonable to believe that OVM-COS-LUT can resist the interference of the gastric environment and stabilize in the intestinal tract. The high stability of OVM-COS-LUT in the alkaline environment also ensured the controlled release and utilization of LUT in the intestine.

### 3.5. Ionic Strength Stability Analysis

**Figure 5:**
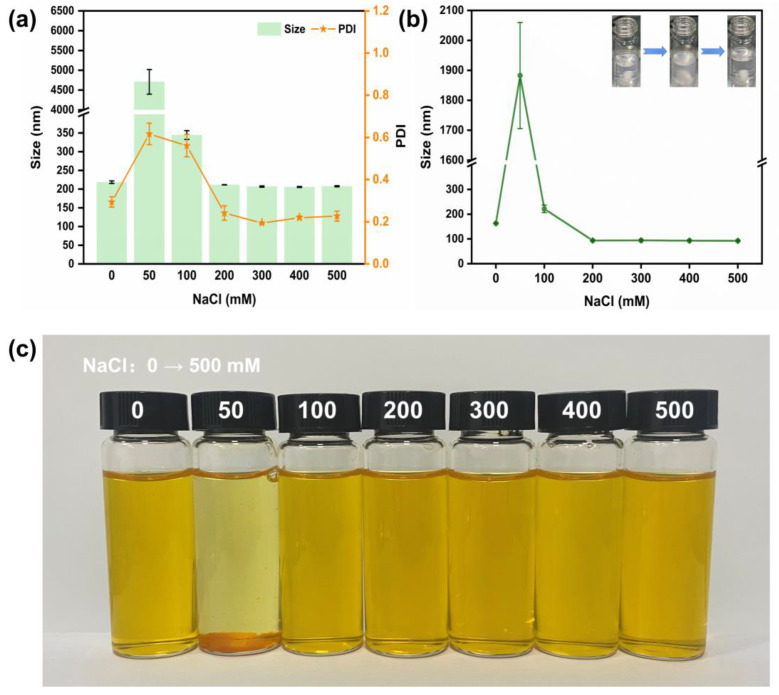
Particle size and PDI of OVM-COS-LUT (**a**). Particle size and solution state changes in OVM (**b**). Optical images of OVM-COS-LUT under different NaCl concentrations (0–500 mM) (**c**).

System stability under various situations, such as ionic strength, is of great importance for cargo-delivery vehicles. When the NaCl concentration was 50 mM, aggregate deposition was observed in the nanogel (Figure 5a,c). The phenomenon above might be attributed to the sharp decrease in the electrostatic repulsion between colloidal particles caused by the electrostatic shielding effect of ions (e.g., Na^+^ and Cl^−^) [42]. With the increase in NaCl addition, the agglomeration tendency gradually degraded. At the concentration of 100 mM, the colloidal particles returned to a steady state of homogeneous dispersion and the particle size exhibited a slight expansion (about 344.53 nm). After the concentration exceeded 200 mM, the average particle size stabilized at about 200 nm and returned to the initial level, which did not change with a further increase in NaCl concentration. In actual gastrointestinal digestion, gastric fluid ionic strength was around 100 mM in the fasting state [43]. After energy intake, a change in ionic concentration >100 mM was often accompanied due to additional external ionic intake. The stabilization of the nanogel at >50 mM ionic concentration contributes to the protection and utilization of the delivered cargo.

In order to further understand the physical properties of OVM and its constructed nanogels, the OVM at different NaCl concentrations was analyzed (Figure 5b). The OVM and OVM-COS-LUT behaved similarly in terms of variation, which was consistent with the structure of the OVM shell of the nanogel. When NaCl concentration approached about 50 mM, the particle size of the OVM increased rapidly, forming larger agglomerates. On the contrary, when the salt concentration covered the threshold, these aggregates were more likely to disintegrate and eventually maintain a stable state. This tendency was also reflected in apparent turbidity. With the increase in NaCl concentration, the OVM presented a clear–turbid–clear change.

### 3.6. Storage Stability Analysis

**Figure 6:**
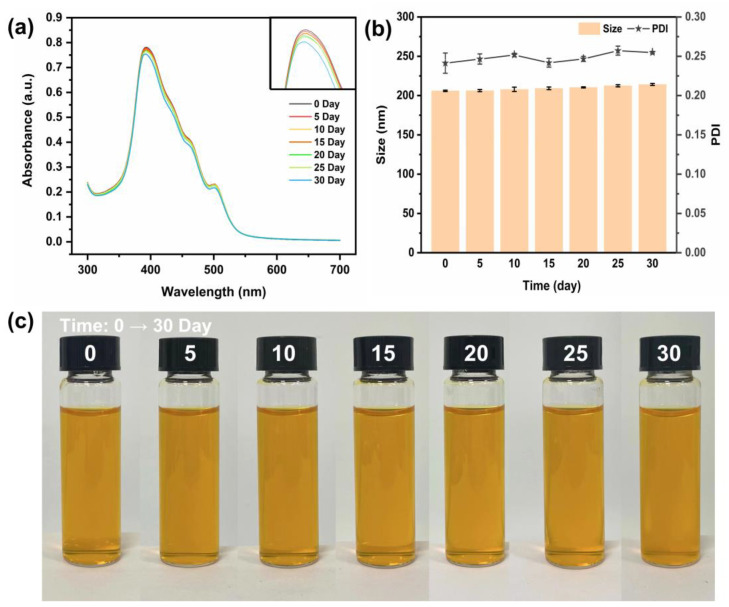
UV–Vis spectrum (**a**); particle size and PDI (**b**); optical images (**c**) of OVM-COS-LUT during 30-day storage.

A principal concern when designing carriers is the protection of functional components within the system. Therefore, the retention of LUT could be a reliable and important index for evaluating the storage stability of OVM-COS-LUT [8]. Due to its unique molecular structure, absorbance measurement could be a reliable way to judge LUT retention. Free LUT is susceptible to oxidative degradation in processing and storage, which is one of the main challenges in achieving effective LUT utilization [44]. As shown in Figure 6a, the absorbance values remained essentially constant during the early stages of the storage period. From 25 days onward, the absorbance began to decrease slightly. After 30 days of storage, the effective retention of LUT by the nanogel was able to reach 96.54%, indicating that the nanogel had long-term LUT protection ability. Meanwhile, in the particle size determination of the nanogel, as shown in Figure 6b, the particle size of OVM-COS-LUT were almost unchanged with the storage time, and the PDI were all kept around 0.25. The results showed that the nanogel had satisfactory storage stability. In addition, the change in macroscopic morphology could reflect the stability of the delivery system, which was usually associated with the interactions between particles. No signs of nanogel deposition were found in the optical photographs (Figure 6c). This indicates that after long-time storage, the colloidal particles could still retain strong interactions to maintain the stability of the system. In summary, OVM-COS-LUT has effective long-term cargo protection and strong storage stability.

### 3.7. Antioxidant and Bioavailability of Lutein

#### 3.7.1. Antioxidant Activity

**Figure 7:**
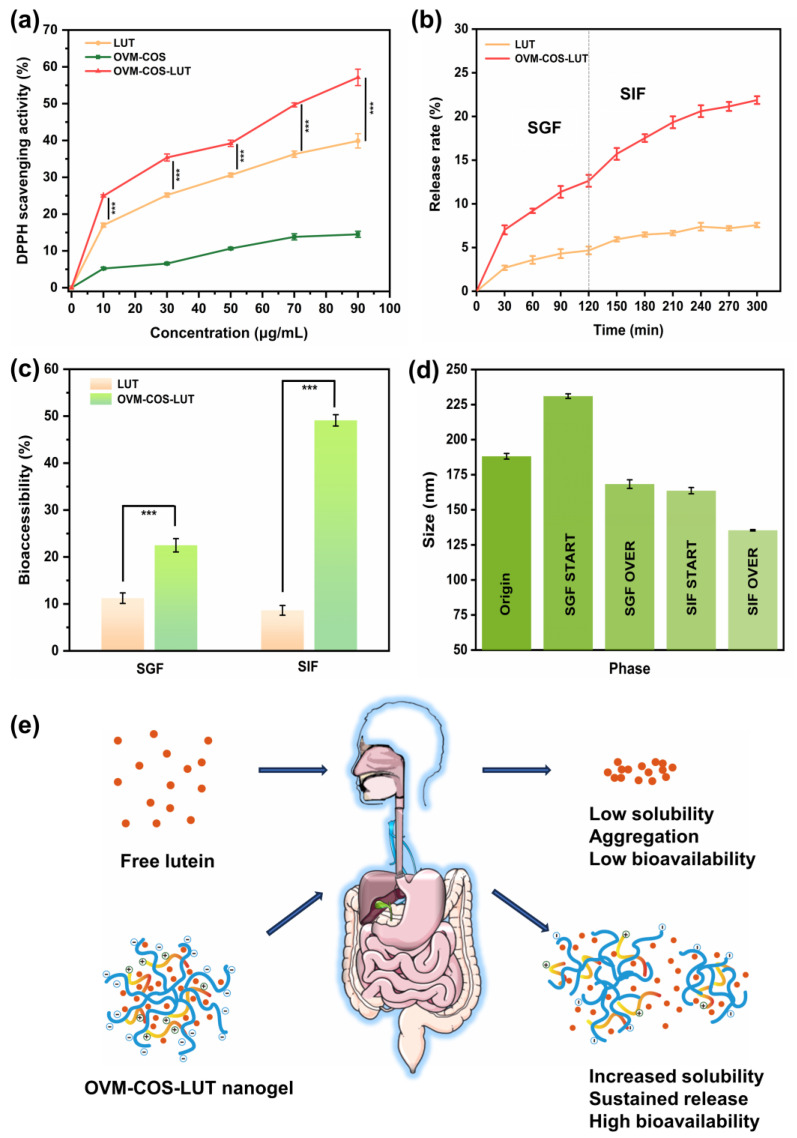
DPPH scavenging activity of LUT, OVM-COS, and OVM-COS-LUT at different lutein concentrations (***: *p* < 0.001) (**a**). Release behavior of LUT and OVM-COS-LUT nanogel in a simulated medium (**b**). Bioaccessibility of LUT and OVM-COS-LUT in simulated gastrointestinal digestion (***: *p* < 0.001) (**c**). Size changes in OVM-COS-LUT in successive gastrointestinal digestion (**d**). Schematic diagram of the simulated release of free lutein and OVM-COS-LUT nanogel (In the schematic structure of OVM-COS-LUT nanogel, the blue line represents OVM, and the gradient red line represents COS.) (**e**). Parts of Figure 7e were drawn using pictures from Server Medical Art. Servier Medical Art by Servier is licensed under Creative Commons Attribution 4.0. https://creativecommons.org/licenses/by/4.0/ (accessed on 15 November 2023).

Antioxidant capacity is considered to be one main bioactivity of LUT. DPPH, a free radical, is widely used to evaluate antioxidant activity in several fields. As shown in Figure 7a, the antioxidant capacity of all samples in different concentrations was analyzed. By contrast, OVM-COS exhibited weaker antioxidant capacity than the two others. It was suggested that the dominant antioxidant capacity from OVM-COS-LUT was not derived from body materials but from the cargo. In addition, under the same LUT level, a significant scavenging differential could be found between OVM-COS-LUT and free LUT (*p* < 0.001). Similar results have been described in a study that prepared a glycosylated zein protein–lutein nanoparticle [45]. The enhancement of scavenging capacity might be explained by the equable LUT dispersibility [46], which was attributed to the binding between LUT and carriers.

#### 3.7.2. Release Behavior

The biodegradation, diffusion, and dissolution of nanoparticles guide the release process, and most drugs have stronger solubility and sustained release ability through nanocarrier encapsulation. As shown in Figure 7b, the release behavior of LUT and OVM-COS-LUT nanogel in a simulated medium was investigated. Free LUT maintained a low release rate throughout the simulated release process, which may be related to its own hydrophobicity. During the release process, free LUT exhibits aggregated deposition, which prevents it from effectively crossing the dialysis bag barrier. By contrast, the release rate of OVM-COS-LUT nanogel for lutein was apparently higher than that of free LUT. The nanogel maintained stable dispersion during the release process, and the enhanced water solubility of LUT allowed for efficient and sustainable release into external media.

#### 3.7.3. Digestion Behavior

In order to understand the behavior of OVM-COS-LUT in complex digestion processing, the effects of the nanogel against degradation in a simulated GI fluid was analyzed through particle size variation. When the nanogel was in SGF incubation, the particle size increased due to component rejection caused by low pH (Section 3.4). The loose structure might have allowed for pepsin cleavage and some LUT to escape, which leads to size reduction (Figure 7d). At the beginning of intestinal digestion, particle swelling was weakened, as the repulsive force was lost due to changes in pH. Perhaps benefiting from the compaction of colloidal particles, the particle size cut dominated by trypsin (163.67 ± 2.23 to 135.40 ± 0.49, about 17%) was weaker than pepsin (231.07 ± 1.59 to 168.33 ± 3.04, about 27%). Meanwhile, compact particles were conducive to the stabilization of LUT, which was ultimately reflected in higher bioaccessibility (26.6% higher than SGF, Figure 7c). Ma et al. found that in in vitro digestion simulations using zein/tea saponin composite nanoparticles to deliver lutein, the nanoparticles had similar results for the improvement of lutein bioaccessibility [33].

According to previous studies, possible physicochemical factors affecting the bioaccessibility of hydrophobic compounds pointed more to their solubility in the gastrointestinal tract [47]. Based on the results, no indication of aggregation and precipitation in nanogel was detected during successive gastrointestinal digestion simulations, which marked the finding that LUT could be stabilized in a dissolved state by wrapping it in nanogel. Related studies have confirmed that precipitation triggered by low water solubility is the main cause of hydrophobic compounds’ low bioaccessibility [47]. From this perspective, the stable dissolution of OVM-COS-LUT throughout the simulated digestion might give it a bioaccessibility far superior to free LUT. Overall, all the phenomena suggested that encapsulation within nanogel could be a practical tool to improve the bioaccessibility of LUT.

### 3.8. Cytotoxicity Analysis

**Figure 8:**
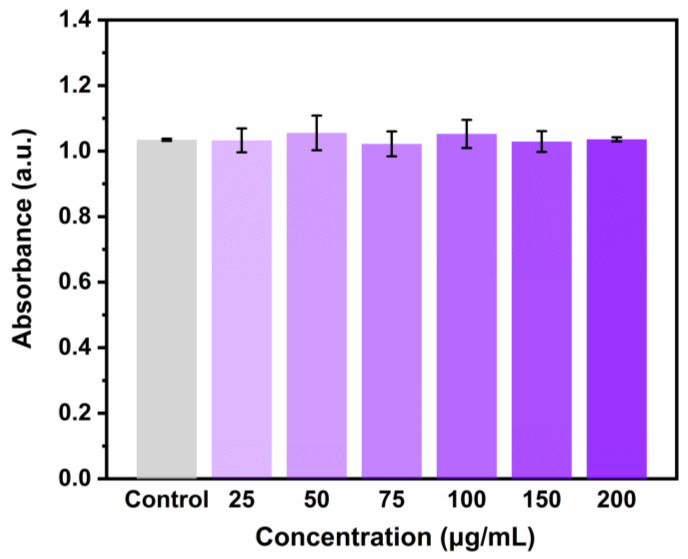
Effect of different concentrations of OVM-COS-LUT nanogel on cell viability (control: no nanogel was added).

Safety is an essential index for evaluating an oral delivery system. The effects of OVM-COS-LUT nanogel on cell viability was measured through the MTT method. In Figure 8, compared to the control group, no statistical difference in absorbance could be found in a wide concentration range. It was suggested that incubation with OVM-COS-LUT was hardly harmful to cell viability. Similar to expectation, the design project formed by natural biomolecules conferred a high safety level on nanogel. Hence, OVM-COS-LUT could be considered a reliable strategy for LUT supplements.

## 4. Conclusions

In this study, nanogel was prepared with OVM and COS to encapsulate LUT in order to improve its bioavailability. The results showed that OVM-COS-LUT appeared to be approximately spherical with a smaller size and narrow particle size distribution. Hydrogen bonding, hydrophobic effects, or other interactions made LUT well encapsulated in a nanogel with an amorphous form, and this enhanced LUT solubility and dispersion, thus exhibiting better antioxidant capacity and bioavailability. In addition, this carrier–cargo interaction helped the nanogel maintain stability in a gastrointestinal environment, enabling the controlled release of LUT. Meanwhile, OVM-COS-LUT also showed good stability in different situations (such as under various pH or ionic strength conditions). Compared with lutein carriers such as liposomes or chemical polymer nanoparticles, OVM-COS-LUT was prepared entirely from natural edible molecules, which gives it a high degree of biocompatibility. Furthermore, its self-assembly method could effectively control the use of organic solvents, reduce the residue of harmful components, and ensure the safety of nanogel. In summary, OVM-COS-LUT could be used as an effective lutein carrier to deliver and improve its stability and bioavailability. This present work presents a simple, efficient, and safe construction strategy for OVM-COS nanocarriers, and it can serve as a reference for applying hydrophobic LUT in the pharmaceutical field.

## Figures and Tables

**Table 1:** Particle size, PDI, zeta potential, EE, and LC of OVM, COS, OVM-COS, and OVM-COS-LUT.

Sample	Size (nm)	PDI	Zeta Potential (mV)	EE (%)	LC (%)
OVM	117.26 ± 2.54	0.44	−26.32 ± 0.38	—	—
COS	3036 ± 614.49	1	+1.95 ± 0.22	—	—
OVM-COS	127.33 ± 2.01	0.29	−20.05 ± 0.31	—	—
OVM-COS-LUT	209.16 ± 1.71	0.25	−20.71 ± 0.68	89.96 ± 0.32	6.47 ± 0.02

**Table 2:** The secondary structure percentage of OVM, OVM-COS, and OVM-COS-LUT.

Sample	α-Helix	β-Sheet	β-Turn	Random Coil
OVM	16.53 ± 0.45 ^a^	30.80 ± 1.80 ^a^	20.23 ± 0.89 ^a^	32.37 ± 0.76 ^a^
OVM-COS	15.83 ± 0.62 ^a^	34.67 ± 0.74 ^b^	18.33 ± 0.49 ^b^	31.13 ± 0.62 ^ab^
OVM-COS-LUT	11.97 ± 0.49 ^b^	43.53 ± 0.84 ^c^	14.36 ± 0.71 ^c^	30.06 ± 0.55 ^b^

Different letters marked on the same column indicate significant differences (*p* < 0.05).

**Table 3:** Zeta potential of OVM-COS-LUT at pH 2–9.

pH	Zeta Potential (mV)
2	26.30 ± 0.47
3	24.33 ± 0.07
4	7.63 ± 0.03
4.5	−3.11 ± 0.44
5	−15.11 ± 0.35
6	−22.66 ± 0.67
7	−28.18 ± 0.55
8	−31.62 ± 0.59
9	−32.65 ± 0.58

## Data Availability

The original contributions presented in the study are included in the article, and further inquiries can be directed to the corresponding authors.
